# Hierarchies of knowledge: ethnobotanical knowledge, practices and beliefs of the Vhavenda in South Africa for biodiversity conservation

**DOI:** 10.1186/s13002-018-0255-2

**Published:** 2018-08-23

**Authors:** Natasha Louise Constant, Milingoni Peter Tshisikhawe

**Affiliations:** 1 SARChI Chair on Biodiversity Value and Change, School of Mathematical and Natural Sciences, University of Venda, Private Bag X5050, Thohoyandou, 0950 South Africa; 2 Sustainable Places Research Institute, Cardiff University, 33 Park Place, Cardiff, CF10 3BA UK; 3 Department of Botany, School of Mathematical and Natural Sciences, University of Venda, Private Bag X5050, Thohoyandou, 0950 South Africa

**Keywords:** Ethnobotany, Indigenous and local knowledge, Traditional ecological knowledge, Biodiversity conservation, Sustainable management, Vhavenda, Knowledge transmission

## Abstract

**Background:**

Indigenous and local knowledge systems are characterised by a ‘knowledge-practice-belief’ complex that plays a critical role for biodiversity management and conservation on indigenous lands. However, few studies take into consideration the interconnected relationship between the social processes underpinning knowledge accumulation, generation and transmission. The study draws on ethnobotanical research to explore plant uses, practices and belief systems developed among the indigenous Vhavenda in South Africa for sustaining indigenous plant resources and highlights some of the forces of change influencing the acquisition and transmission of knowledge.

**Methods:**

Data was collected from September–November 2016 from 31 individuals by means of semi-structured interviews; walks in home gardens, cultivated fields, montane forests and deciduous woodlands; and vouchering of plant species in six villages (Duthuni, Tshidzivhe, Vuvha, Lwamondo, Mashau and Tshiendeulu) in the Vhembe District of South Africa. The Use Value Index (UVI) was used to measure the number of different uses of each species and the Relative Frequency Index (RFI) to measure the local importance of each species. Semi-structured interviews and comparisons with published works also explored cultural practices and belief systems associated with plants, modes and barriers of knowledge transmission.

**Results:**

Eighty-four plant species were reported within 44 families, with Fabaceae representing the highest diversity of plant species. We identified six species not previously documented in the Vhavenda ethnobotanical literature, 68 novel uses of plants and another 14 variations of known uses. Vhavenda plants were predominantly used for food (36.0%) and medicine (26.1%) and consisted mainly of native (73.8%) compared to non-native species (26.2%). The Vhavenda possess a range of practices for managing plant resources that can be attributed to taboos preventing the use of selected species, promotion of sustainable harvesting practices and the propagation of plant species for ecological restoration. Plant knowledge and management practices were transmitted from relatives (48.4%), self-taught through time spent planting and harvesting plants on the land (19.4%), through apprenticeships with traditional healers (16.1%), initiation schools (9.7%) and clan gatherings (6.4%). Changes in traditional learning platforms for knowledge exchange, erosion of cultural institutions and shifting value systems serve as barriers for knowledge transmission among the Vhavenda.

**Conclusion:**

The study points to a need for new partnerships to be forged between conservationists, government actors and local and indigenous knowledge holders to foster hybrid knowledge coproduction for developing strategies to enhance the productivity and biodiversity of indigenous lands.

## Background

Indigenous and local communities have devised cultural practices embedded in cultural and religious values that have maintained species and habitats of biocultural importance through indigenous and local knowledge systems [1]. Indigenous and Local Knowledge (ILK) consist of a body of knowledge shaped by cultural practices, institutions and worldviews forming a nested ‘knowledge-practice-belief’ complex that provide insights into ways of knowing and governing social-ecological systems (SESs) for contemporary biodiversity management and conservation [1]. Indigenous plants provide a plethora of ecosystem services to support human needs for food, medicines, livelihoods and other cultural activities [2]. Plant resources are sustained through cultural practices where plant users collect and harvest materials selectively using locally adapted management strategies [3], that is also important for the conservation of biodiversity, rare species, ecological processes and sustainable harvesting practices [1, 4]. The application of biodiversity management through cultural practices can help to strengthen cultural values compatible with conservation to sustain plant resources for biodiversity and to support human needs [5]. In recognition of this, many international and national agencies and agreements (e.g. Convention of Biological Diversity (CBD), Intergovernmental Platform on Biodiversity and Ecosystem Services (IPBES)) advocate for enhanced engagement and protection of the customary use of biogenetic resources in accordance with cultural practices to promote biodiversity conservation and sustainable use of natural resources.

Cultural practices are embedded in institutions and local social norms that influence the coordination and management of resource use practices often through traditional leaders who control access and use of natural resources on indigenous lands [4]. Taboos play a role in limiting destruction of important plant resources through limitations on harvesting and prohibitions against the use of selected species. Rituals, ceremonies and other traditions associated with plant use serve to pass on institutional memory and cultural internalisation to support knowledge generation, accumulation and transmission [4]. Similarly, worldviews are important for delineating beliefs, and cosmologies that influence cultural values, and ethics for engaging with the natural world [4]. These social mechanisms are critical for understanding how institutions and norms structure and transmit plant-based knowledge and influence modes of knowledge transmission to support the revival of sustainable management practices for biodiversity conservation.

The aim of the study is to examine the use, cultural practices and beliefs associated with plant species among a rural community in South Africa for biodiversity conservation on communal lands using a case study of the Vhavenda. Ethnobotanical research on the Vhavenda has focused on people’s use and knowledge of indigenous plants [6], plants for food [7], nutrition [8], medicine [9–11], the treatment of livestock diseases [12], beverages [13], invasive species [14, 15] and impacts of bark harvesting [10]. Some studies have addressed the role of Vhavenda culture and conservation of the wider bio-physical environment [16] and the protection of sacred sites [17]. However, few studies have examined the interconnected relationships between plant-based knowledge, practices and beliefs of diverse plant species for biodiversity conservation and the nature and barriers of knowledge transmission in the context of social-cultural change. The hypothesis of the study is that the Vhavenda exhibit a complex ‘knowledge-practice-belief’ system of ILK that acts to sustain plant resources and carries insights to inform culturally specific management strategies for the conservation of indigenous plants on community lands. The study aims to address the following questions: (1) What are the characteristics of plant species used by the Vhavenda and which species are most important in terms of their use value and frequency of use? (2) What cultural practices, institutions and beliefs sustain plant resources for biodiversity conservation? and (3) How is plant-based knowledge acquired and what are the barriers for the transmission of knowledge to younger generations?

## Methods

### Vhavenda history and social structure

Oral accounts of the early history of the Vhavenda prior to their entry from present day Zimbabwe are vague and fragmented; however, it is probable that their origins were located in the Great Lakes region of East Africa [18]. The Vhavenda are composed of different clans, for example, Senzi, Nyai, Mbedzi, Lemba, Ngona, Ludzi, Kwevho, Nzhelele, Luvhu, Famadi and other smaller nuclear groups [19]. The first Europeans arrived in Venda during the early 1800s including missionaries, explorers, hunters and land speculation companies. Contestations between the Vhavenda and the first colonisers (named the Voortrekkers) ensued over conflicts of land and natural resource resulting in ongoing wars between the late 1880s–1900s [20]. The first missionary church was established in the region named Schoemansdal in 1851 [17]. The missionaries acted as neutral entities during the earlier colonial wars but asserted their own form of colonisation through the erosion of traditional cultural practices and belief systems of the Vhavenda.

The onset of apartheid headed by the National Party saw the establishment of the Bantustans where South Africans were segregated by ethnicity to create territories and ‘autonomous’ national states [17]. The Prime Minister of the time Verwoerd sought to reduce the number of Africans resident in South Africa’s urban areas by requiring them to live in their respective homelands and to seek employment in nearby towns and cities to address rural poverty [21]. Vhavenda chiefs who were ruling at the time favoured the new government policy mainly because they were able to maintain their traditional system of governance [22]. In 1973, Vhavenda was granted self-government under the leadership of Chief Patrick Mphephu who traced his ancestry to the legendary leader Thohoyandou [23].

The current social structure of the Vhavenda chieftainship is headed by the *Thovhele* meaning ‘king’ who rules over the largest regions of Vhavenda [21]. The *Khosi*, a ‘senior chief,’ rules an area of more than two villages, and under his jurisdiction, each village is ruled by a *Vhamusanda*, a junior chief [19]. A Vhamusanda may appoint a *Mukoma* who settles disputes in the village and is responsible for allocating arable land to individual homesteads, organising social rituals, and the protection of the natural environment [6]. The *Makhadzi* is usually a position held by one of the *Khosi’s* sisters, but only one sister can be chosen by the royal council who serves as an advisor to the *Khosi*, resolves conflicts within the royal family and participates in ritual ceremonies and rites of passage [19]. The traditional religion of the Vhavenda is based on the belief of a supreme being named *Nwali* who influences the world of the living by identifying his presence in storms, rains and earthquakes and is appeased through ritual ceremonies [19]. The Vhavenda believe that the deceased are taken by the ancestors, who continue to exert control over the lives of the living by bringing peace or ill fortune and are venerated and appeased through ritual practices [6, 19]. The *Makhadzi* and traditional healers serve to maintain a link between the dead and the living and are responsible for different rituals involving libations and prayers in veneration of ancestor spirits. Although these rituals may serve as a family affair, the *Makhadzi* of the royal family often serves as a representative for the whole of the royal ancestors and will perform public ceremonies such as the first fruits and vegetable ceremonies [19].

### Study site

**Fig. 1:**
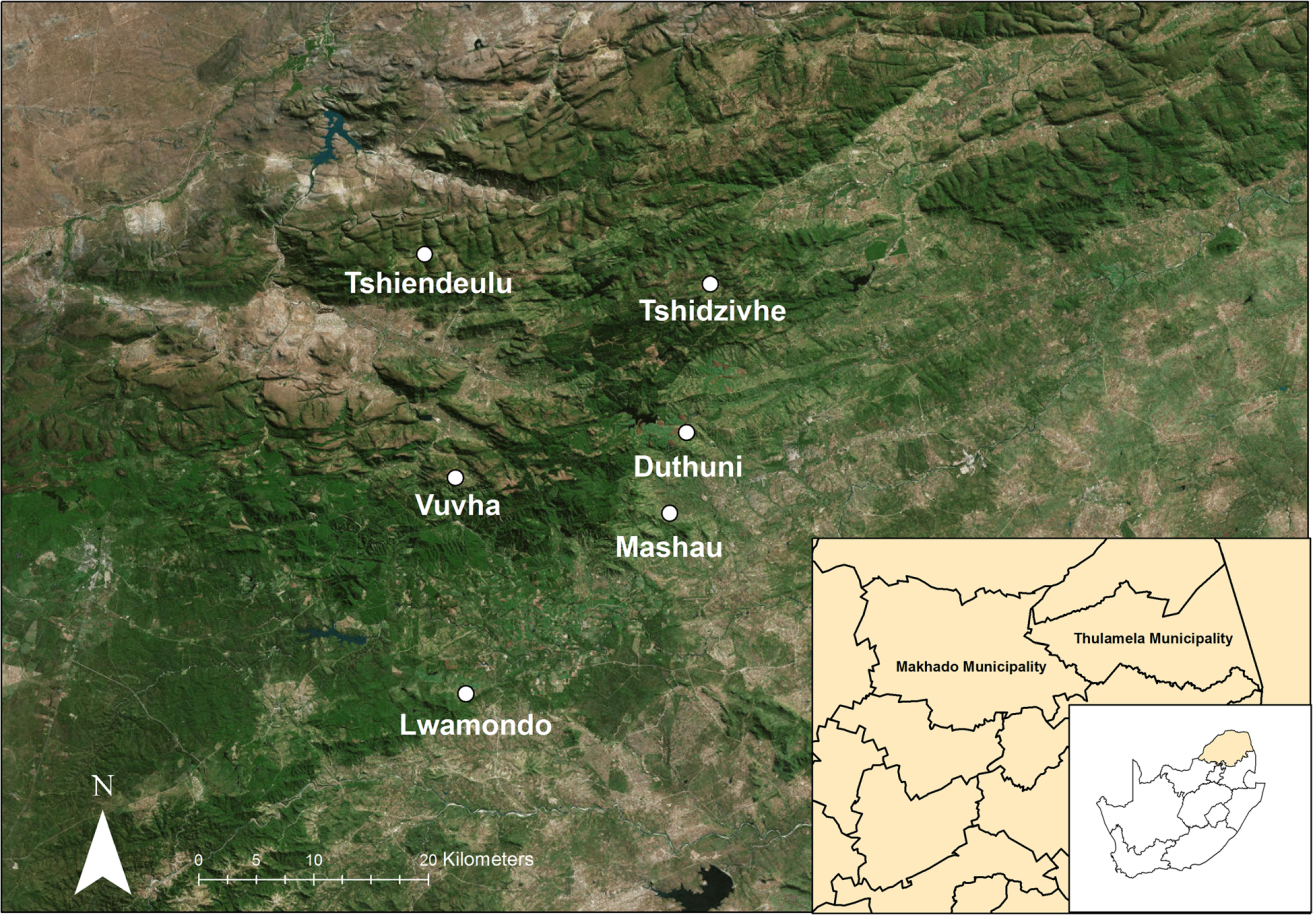
Map of sampled villages of the Vhavenda community, Limpopo Province, South Africa

The study took place in six villages (Duthuni, Tshidzivhe, Vuvha, Lwamondo, Mashau and Tshiendeulu) located in the Vhembe District of the Limpopo Province, South Africa, and forms part of the eastern Soutpansberg Mountain Range in the Vhembe Biosphere Reserve (Fig. 1). The villages of Duthuni, Tshidzivhe and Lwamondo are located in the Thulamela Municipality and Vuvha, Tshiendeulu and Mashau in the Makhado Municipality (Fig. 1).

The area is characterised by a subtropical climate with moist winters and wet, warm summers [24]. The area experiences an annual rainfall of 500 mm of which 87% of rainfall occurs between October and March [24]. Annual temperatures range from 10 °C in winter to a maximum of 40 °C in the summer [24]. The Soutpansberg Mountain Range is a centre for botanical endemism in southern Africa and falls under the mountain bushveld vegetation unit of the Savannah biome that includes dense deciduous woodlands and evergreen montane forests and open savannah in places [25]. The southern slopes of the eastern portion of the Soutpansberg Mountain support dense deciduous woodlands at lower altitudes consisting of small-tree species (dominated by *Diospyros whyteana* (Hiern) P. White, *Englerophytum magalismontanum* (Sond.) T. D. Penn, and *Schefflera umbellifera* (Sond.) Baill) and dense evergreen montane forests consisting of small-tree species (e.g. *Brachylaena transvaalensis* Hutch. ex E. Phillips & Schweick, *Celtis africana* Burm. f, *Cussonia spicata* Thunb) [25, 26]. The more arid northern ridges consist of tall-trees (e.g. *Acacia nigrescens* Oliv, *Adansonia digitata* L, *Brachystegia spiciformis* Benth) and small-tree species (e.g. *Combretum apiculatum* Sond, *Commiphora glandulosa* Schinz) [26, 27]. Venda’s landscapes have also undergone significant land-use change through the establishment of commercial forestry and tea plantations during the 1930s–1970s to provide economic development in the Venda homeland. The confinements of the former Venda homeland in the semi-urbanised villages surrounding the town of Thohoyandou necessitate a demand for housing, and clearing of land for agriculture has resulted in the degradation and fragmentation of montane forests and deciduous woodlands that are largely confined to river valleys and higher elevations of the mountain on south-facing slopes [27].

### Data collection

Data was collected from September–November 2016 by working in collaboration with the community-based organisation Dzomo La Mupo who acted as a key gatekeeper to access the targeted villages. Initial meetings with communities were established in consultation with the Chief (*Khosi*) or headman (*Mukoma*) to describe the aims and objectives of the study, identify informants who were knowledgeable of plants and seek permission for the study. Each participant was presented with an information sheet and consent form (approved by the Research Ethics Committee of Cardiff University) which clarified the objectives of the study, and all informants were asked to sign consent forms to secure informed consent to participate in the study. Semi-structured interviews were conducted in the local language (Tshivenda) through the assistance of a translator and later translated into English. Questions focused on the important plant uses including their local names, habits of the plant, parts used, collection sites and plant uses. Local practices and belief systems associated with the management and protection of plant resources were explored. Finally, discussions with all informants addressed perceptions of how plant-based knowledge is transmitted and the perceived barriers for knowledge transmission. Selection of study participants was made using a snowball sampling strategy in consultation with the *Khosi* or *Mukoma* from each village to identify individuals for interview based on the condition that they were knowledgeable of plants or were specialists in the community, for example, traditional healers. A total of 31 people were interviewed, namely 18 females (58.0%) and 13 men (42.0%), ranging in age from 34 years to 85 years. Of all informants, 35.5% received no formal education, 29.0% received a primary school education, while 35.5% received a secondary school education.

### Plant collections

Collections of plant specimens were obtained by walking in home gardens, cultivated fields, montane forests and deciduous woodlands. During collection, the species was classified according to its habit: tree, shrub, herbaceous, climbing or grass [28]. Plants were later classified according to their biogeographical region to determine whether they were native or exotic plants. The plant species were identified by PT with the aid of the literature and comparison with herbarium species, and voucher specimens were prepared and deposited at the University of Venda Herbarium (UVH) in South Africa. Plant species were categorised as ‘invasive,’ ‘naturalised’ and ‘casual alien’ classifications [29, 30] and by drawing on species records of alien plants using the Southern African Plant Invaders Atlas (SAPIA) [31].

### Data analysis

Descriptive statistics such as frequencies and percentages were used in the analysis of the data. The Use Value Index (UVI) and the Relative Frequency Index (RFI) were calculated to determine the most important plant species sampled [32]. The UVI is a measure of the relative importance, measured as the number of different uses of each species: UV *= ΣUi/N*. It is calculated by the total number of uses of a plant mentioned by a participant (*U*) divided by the total number of participants in the study. The RFI is an index of the local importance of each species: RFI = FC/*N* in which the FC is the number of participants that mention the use of a species divided by the total number of participants in the study.

## Results

### Indigenous plants of the Vhavenda

There are a total of 84 plant species in the sampled communities all of which were identified to species level from 44 families (Table 1). The best represented families were Fabaceae with ten species and Apoynaceae, Asteraceae, Combretaceae, Cucurbitaceae and Rubiaceae all with a total of four species. The other families (64.3%) were represented by one to three species.

**Table 1:** Plant species cited by informants among Vhavenda communities in the Limpopo Province of South Africa

Scientific and family name	Vhavenda name	Habit	BO	Status	CS	AP	P	Use value	Description	Literature on Vhavenda ethnobotanical use	Voucher numbers	UVI	RFI
Acanthaceae													
Ribbon Bush (*Hypoestes aristata* (Vahl) Roem. & Schult.)	Mukuluvhali	H	N	C/W	HG		Y	Food, medicine	a) Leaves: eaten as a vegetable; relish eaten with porridge; b) *Roots: menstrual pain; bolsters immunity in young babies*^*1*^	a) Leaves: vegetable; eaten during times of food shortage [6].	NCU0014	0.065	0.032
Amaranthaceae													
Fat Hen, Lamb's Quarter, White Goosefort, Common Pigsweed (*Chenopodium album* L)	Dale Dale	H	E (Europe)	W	HG	C/N		Food	a) Leaves: edible vegetable; relish seasoned with salt and eaten with porridge; dried in the sun and stored.	a) Leaves: vegetable [6].	NCU0065	0.032	0.032
Red amaranth, Wild amaranth, Purple Amaranth, Cockscomb (*Amaranthus cruentus* L)	Mukango	H	E (North and Central America)	W	HG			Food, cultural, charcoal	a) Leaves: edible vegetable; eaten with porridge; b) *Stem: seasoning to be mixed with the leaves of Corchorus tridens*; *also mixed with Ricinus communis to make a stimulant named snuff*^*1*^; c) *Bark stem: used to make charcoal*^*1*^**.**	a) Leaves and stem: edible vegetable served with other vegetables and pumpkin leaves; dried and stored for future use [7].	NCU0021, 117, 135	0.097	0.097
Smooth Pigweed (*Amaranthus hybridus* L)	Vowa	H	E (America)	C/W	HG	C/N	Y	Food, medicine	a) Leaves: edible vegetable; mixed with tomatoes and salt; relish eaten with porridge; infusion of leaves used to make a drink for new-born children named *khongodoli to bolster the immune system*^2^; *infusion of leaves is also used to treat high blood pressure*^1^*.*	a) Leaves: vegetable eaten with pumpkin leaves and flowers and eaten with *Corchorus tridens*; used to test suitability of baby’s food during first 3–4 days. If the baby shows symptoms of diarrhoea, it is given a soft porridge named *khongodoli* instead of *ntsu* a liquid food. In this case, the baby is given a decoction of boiled leaves; ingredient in snuff; high nutritional value [6–8].	NCU0049, 94, 114, 118, 125, 157,	0.065	0.226
Anarcardiaceae													
Marula (*Sclerocarya birrea* (A.Rich.) Hochst.)	Mufula	T	N	C/W	F/W		Y	Firewood, food, medicine, drink	a) Fruits: edible and used to make a beer named *mukumbi*; b) Bark: treat ulcers; supports pregnancy and fertility in women; c) Wood: firewood.	a) Fruit: edible fruits; beer; b) seeds: cooking fat; cooking oil; c) bark: support pregnancy; fertility; colds; headaches; malaria; stomach troubles; ulcers; toothache; regulate sex of unborn child; d) wood: for carving and household utensils; burning articles made from clay [6, 9, 16].	NCU0140, 158, 176	0.129	0.097
Annonaceae													
African Custard Apple, African Custard Apple (*Annona senegalensis* Pers)	Muembe	T	N	W	HG			Firewood, food, medicine	a) Fruit: edible fruits; b) Bark: *toothache*^1^; root bark added to porridge for babies for its nutritional value; c) *Wood: firewood*^1^**.**	a) Fruit: edible fruits; b) Root: snake-bite; venereal disease; bilharzia; enhances medicinal and nutritional value of porridge; relieves constipation; stomach and spasms; headache due to indigestion; blood in faeces; c) Bark: stomach ache; diarrhoea; dysentery; protecting individuals from antagonistic individuals; fibre used to make ox-whips; d) Wood: cow-stick; e) Branch: headache [6, 9, 16]	NCU0180	0.097	0.032
Kalahari Bitterwood, Kalahari Red-Fingers (*Xylopicrum odoratissimum* (Welw. ex Oliv.) Kuntze)	Muvhulavhusiku	T	N	W	HG			Medicine	a) Roots: stomach pain	a) Roots: stomach ache [6, 16].	NCU0082	0.032	0.032
Apocynaceae													
African Heartvine (*Pentarrhinum insipidum* E.Mey.)	Phulule	C	N	W	HG			Food	a) Leaves: edible vegetable; cooked with porridge.	a) Edible vegetable; used as a spice when cooked with other vegetables [6].	NCU0070	0.032	0.032
Quinine Tree (*Rauvolfia caffra* Sond)	Munadzi	T	N	C	HG		Y	Medicine, shade, domestic utensils	a) Wood: household utensils such as spoons and bowls; b) Bark: stomach aches; c) *An important shade tree*^1**.**^	a) Bark: killing maggots in wounds; abdominal and pelvic troubles; malaria; arrests development of diseases; epilepsy; b) Wood: household utensils such as spoons and bowls [6, 9, 49].	NCU0013	0.097	0.032
Rubber Vine (*Landolphia Kirkii* Dyer)	Muvhungo	Shr	N	W	HG			Food	a) Fruits: edible fruits named *muvhungo*; b) Latex: strengthening birdlime.	a) Fruits: edible fruits; beverage; b) Latex: sweet taste and used to make birdlime; c) Roots: piles; rheumatoid arthritis; d) Stem: sticks to protect against witchcraft and magical attacks; e) Saplings: basket rims; constructions of thatch roofs [6, 9, 13].	NCU0085, NCU0194	0.032	0.065
Simple-Spined Num-Num, Climbing Num-Num, Small Num-Num (*Carissa edulis* (Forssk.) Vahl)	Murungulu	T	N	W	HG			Firewood, food	a) Fruit: edible fruits; b) Roots: soaked and mixed with other vegetables to make a relish and eaten with porridge; c) *Wood: firewood*^1^	a) Fruits: edible fruits; juice; b) Roots: mixed with other roots to make an infusion for soft porridge named *tshiunza* and eaten by babies; tuberculosis; menorrhagia; infertility; worms; increase size of penis, mild laxative for children; c) Leaves: stomach ache; cough; cataracts [6, 9, 13, 50].	NCU0174, 201	0.065	0.065
Aracreae													
Taro (*Colocasia esculenta* (L.) Schott)	Mufungwe	H	E (Asia)	C/W	HG	C/N		Food	a) Leaves: edible vegetable, leaves are dried and stored for future use; *often eaten with meat because it produces an attractive aroma*^2^. The plant is commonly found in rivers.	a) Leaves: edible vegetable; b) Rhizomes: taste like potatoes when fried [7].	NCU0002, 19, 68, 92, 99, 144	0.032	0.226
Araliaceae													
False-Cabbage Tree (*Schefflera umbellifera* (Sond.) Baill)	Mukho	T	N	C/W	HG		Y	Firewood, construction	a) Wood: used to make household utensils such as spoons and plates; firewood.	a) Wood: household utensils such as knives; spoons; plates; bowls [6].	NCU0053	0.065	0.032
Asteraceae													
Annual Sowthistle, Common Sowthistle (*Sonchus oleraceus* (L.) L.)	Shashe	H	E (Europe, Asia)	W	HG	C/N		Food	a) Leaves: edible vegetable believed to *bolster the immune system*^2^	a) Leaves: piquant taste to cooked vegetable; dried and stored for future use [6].	NCU0152	0.032	0.032
Black Jack, Beggars Ticks (*Bidens pilosa* L)	Mushidzhi	H	E (America)	W	HG	C/N	Y	Food, medicine	a) Leaves: edible vegetable eaten with porridge; *leaves are dried and used during times of drought or famine*^2^; *bolsters the immune system*^1^; given to new born babies to be eaten with soft porridge.	a) Leaves: edible vegetable eaten with porridge; piquant with other vegetables; menstruation problems; promote conception; testing whether new-born babies need liquid food or solid porridge; high nutritional value [6–8].	NCU0022, 60, 62, 95, 126, 151		
Bushman’s Tea (*Athrixia phylicoides* DC)	Mutshatsha	Shr	N	W	HG		Y	Food, drink	a) Leaves: *Boiled and mixed with Hibiscus trionum to make a tea*^2^; b) *Fruits: used to make a dish named thopi from the fruits named gwadi*^1^*.*	a) Root: aphrodisiac; anthelmintic; b) Leaves: tea named *Mubosotie*; meaning wild tea plant; c) Wood: broom; d) Other uses: heart disease; diabetes; high blood pressure; headaches; stomach aches; influenza; leg wounds [6, 13, 51].	NCU0187	0.065	0.032
Forest Silver Oak (*Brachylaena discolor* DC)	Mufhata	T	N	C/W	HG		Y	Firewood, domestic utensils, construction, charcoal, medicine, crafts, cultural	a) Wood: carving spoons; fences; huts; and poles; *support for the neck to correct bad posture in children*^1^; *threads for ties*^1^; firewood; *charcoal*^1^; b) Leaves: treating roundworm; c) tree found in rivers; forests and mountains.	a) Leaves: roundworm infection; b) Wood: roofs; fencing; posts; wall posts; tool handles; firewood [6].	NCU0054, 57	0.226	0.097
Athyriaceae													
Lady Fern (*Athyrium filix-femina* (L.) Roth)	Muvangulure	Shr	E (North America)	W	HG			Indicator	a) *Grows in cultivating fields and its presence indicates soil fertility*^1^	No known records	NCU0177	0.032	0.032
Capparaceae													
African Cabbage, Spider Wisp (*Cleome gynandra* L)	Murudi	H	N	W	HG		Y	Food	a) Leaves: edible vegetables; the leaves are cooked and eaten as a relish with tomatoes and served with porridge; leaves can also be dried and stored during winter.	a) Leaves: edible vegetable; eaten with porridge; spice favoured for its piquant taste; dried and stored for future use; high nutritional value [6–8].	NCU0131, 184	0.032	0.129
Caricaceae													
Papaya tree (*Carica papaya* L.)	Mupapawe	T	E (Central America)	C	HG	C/N	Y	Food, commercial, medicine	a) *Fruits: Face mask to clear pimples*^1^; *body lotion*^1^; edible fruits; b) *Bark: steam of bark used to cure symptoms of flu*^1^	a) Roots: venereal disease [9].	NCU0073, 100, 147	0.097	0.097
Chrysobalanaceae													
Cork Tree (*Parinari curatellifolia* Planch. ex Benth.)	Muvhula	T	N	W	HG			Food, drink	a) The fruits are eaten when ripened and an alcoholic beverage is also made from the fermented pulp of the fruit.	a) Fruits: for edible fruits; stamped in water or milk; alcoholic beverage; b) Bark: pelvic pains; venereal diseases; cleaning kidneys; toothache; c) Roots: venereal disease [6, 9, 13].	NCU0084	0.065	0.032
Combretaceae													
Bicoloured Bushwillow, Kalahari Bushwillow, Silver Bushwillow (*Combretum collinum* Fresen)	Muvuvha	T	N	W	HG			Firewood, charcoal	Wood: firewood; *charcoal*^1^	a) Wood: firewood; b) Shade saplings: building material [6].		0.065	0.032
Bush Willow, Bushveld Willow (*Combretum erythrophyllum* (Burch) Sond)	Muvuvhu-wa-mulamboni	T	N	C	HG		Y	Medicine	a) Bark: pregnancy problems; b) Tree is found close to rivers.	a) Bark: pregnancy problems; b) Branches: roofs and wattles; c) Roots: coughs [6, 9].	NCU0105	0.032	0.032
Silver-cluster leaf (*Terminalia sericea* Burch. ex DC.)	Mususu	T	N	W	HG			Medicine	a) Roots: treats diarrhoea in young babies.	a) Roots: used in soft porridge to prevent diarrhoea and dysentery; arrest purging; treat protracted parturition or a hanging placenta; venereal disease; infertility [6, 9].	NCU0124	0.065	0.032
Velvet Bush Willow, Velvet Leaf Willow (*Combretum molle* R.Br ex G. Don)	Mugwiti	T	N	C/W	HG, CL			Firewood, charcoal, medicine	a) *Bark: diarrhoea*^1^; b) Leaves: common colds; c) Wood: firewood.	a) Wood: firewood; construction; building; b) Leaves: colds; c) Medicine to encourage and maintain pregnancy; Roots: laxative [6, 9].	NCU0040, NCU0179	0.097	0.065
Cucurbitaceae													
Balsam Pear (*Momordica balsamina* L)	Tshibavhe	C	N	W	HG			Food, medicine	a) Leaves: edible vegetable; leaves of plant; eaten with porridge; *high blood pressure*^1^	a) Leaves: edible vegetable eaten with porridge; piquant taste when added to other vegetables; antiemetic [6].	NCU0063	0.065	0.032
Bitter Melon (*Momordica boivinii* Baill)	Nngu	C	N	W	HG			Food, medicine	a) Leaves: edible vegetable; eaten during times of drought; b) Leaves and roots: *Earache*^1^; *gout*^*1*^	a) Leaves: eaten with porridge; spice; b) Roots: helps babies to grow bigger [6, 7].	NCU0067, NCU0156	0.097	0.065
Jelly Melon, Bitter Wild Cucumber, African Cucumber (*Cucumis africanus* L.f.)	Tshinyagu	H	N	W	HG			Food	a) Leaves: *Edible leaves and mixed with Hibiscus Trionum to be eaten with porridge*^2^.	a) Leaves: edible vegetable; b) Seed: purgative [6, 7].	NCU0186	0.032	0.032
Pumpkin (*Cucurbita pepo* L)	Thanga	H	E (Central and North America)	C	HG		Y	Food, medicine	a) Leaves: edible vegetable; eaten as a spinach when cooked with the roots; eaten with porridge; *medicine for treating birth pain*^1^ b) Seeds: edible; c) Calabash; d) *Skin of pumpkin: used to make an edible dish named thopi when mixed with porridge*^2^*.*	a) Leaves: cooked with pumpkins and flowers as a dish; b) Flowers: dried and used as a vegetable [6, 7].	NCU0076, 103, 134, 182, 191	0.065	0.161
Ebenaceae													
Bluebrush, Star-Apple Monkey Plum (*Diospyros lycioides* Desf)	Muthala	T	N	C/W	HG			Firewood, Food, Shade	a) *Fruits: salads*^1^; *Wood: firewood*^1^	a) Roots: used to make *tshiunza* a dish given to babies with porridge and *ntswu*; a nutritious fluid of plants used to feed children who cannot yet eat soft porridge; epilepsy b) Stems: used as lashes by herd boys and teachers [18, 21].	NCU0026, 148, 170	0.097	0.097
Magic Guarri (*Euclea divinorum* Hiern)	Mutangule	T	N	W	HG			Firewood, medicine, food	a) Fruits: edible fruits; b) Branches: toothbrush; c) *Wood*: *firewood*^1^; d) Roots: stomach problems.	a) Fruits: edible fruits; beverage; b) Branch: toothbrush; c) Roots: purgative; stomach aches; purification of blood; general ill health; c) Prevent water contamination [6, 13, 16, 52, 53].	NCU0168	0.097	0.032
Euphorbiaceae													
Cassava (*Manihot utilissima* Pohl)	Mutumbula	Shr	E (South America)	C	HG			Food	a) Leaves: cooked with soft porridge for babies; *aids digestion*^*1*^; eaten during times of hunger or drought.	a) Leaves: edible vegetable; eaten with porridge and stored for future use; b) Roots: tuber eaten after prolonged boiling or central root core is removed prior to cooking as it is believed to be poisonous [6].	NCU0102	0.032	0.032
Castor Oil (*Ricinus communis* L*.*)	Mupfure	H	E (Europe, India, Tropical Africa)	C	HG	I		Medicine	a) Seeds: oils mixed with other medicines because of sticky substance; polishing leather; (b) *Leaves: dried and crushed to make a snuff*^*1*^*.*	a) Roots: toothache; (b) Leaves: purgative; used to treat the disease *tshiliso*; thought to be caused by witchcraft; topical treatment of internal pains and injuries; c) Seed: purgative; oil from the seed made for mixing medicines; earache; softening and polishing leather; d) Fruits: used as slingshot balls; causes diarrhoea and emesis but will cure coughs; worms; laxative; tonic; earache; menorrhagia; f) Leaves and stems: stings; bites of insects [6, 9, 14].	NCU0051	0.032	0.032
Forest Fever Berry (*Croton sylvaticus* Hoscht)	Mulathoho	T	N	C/W	HG		Y	Shade, medicine, firewood	a) *Shade*^1^; b) *Leaves: pleurisy*^1^; c) *Wood: firewood*^*1*^*.*	No known records	NCU0047	0.097	0.032
Fabaceae													
Ana Tree, Apple Ring, Winter Thorn(*Faidherbia albida* (Delile) A. Chev)	Muhoto	T	N	C	HG		Y	Food, medicine	a) *Fruits: eaten by cattle*^1^; b) *Bark: venereal disease*^1*.*^*.*	a) Bark: anti-malarial [10].	NCU0104	0.065	0.032
Apple-Leaf (*Philenoptera violacea* (Klotzsch) Schrire)	Mufhanda	T	N	C	HG			Firewood, medicine, c	a) *Stems: medicine for protecting the homestead and yard*^1^; diarrhoea; b) *Wood: firewood*^*1*^*.*	a) Bark: treatment of ticks; b) Entire plant: diarrhoea; c) Roots: gastrointestinal disorders [9, 54].	NCU0120	0.129	0.032
Common Coral Tree, Lucky Bean Tree (*Erythrina lysistemon* Hutch)	Muvhale	T	N	C/W	HG			Construction, medicine, cultural, food	a) Fruit: edible fruits; b**)** *Wood: fences for construction*^1^; c) *Bark: enhance the immune system*^1^; d) Cultural: tombstone for traditional graveyards	a) Planted in graveyard; b) Bark: toothache; antibacterial compound; improved sexual performance; relieving oedema; c) Wood: firewood; d) windbreak; e) ornamental [6, 10, 11, 16].	NCU0029, 164	0.129	0.065
Cork bush, Silver Bush, Rhodesian Silver-Leaf (*Mundulea sericea* (Willd.) A. Chev.)	Mukundandou	T	N	W	HG			Firewood, medicine	a) Roots: protection against witchcraft; b) Wood: firewood	a) Roots: protection against witchcraft; aphrodisiac; to regulate sex of unborn child; b) Strong medicine to evade or subdue; kunda = to conquer + ndou = elephant referring to the strongest animal; c) Wood: firewood [6, 9].	NCU0042, NCU0081	0.065	0.065
Cowpeas (*Vigna unguiculata* (L.) Walp)	Munawa	H	N	C	HG		Y	Food, medicine	a) Leaves: edible vegetable; relish eaten with porridge; b) Fruits: eaten with soft porridge and mixed with jugo beans; groundnuts; maize which has been grounded; and the powder of grounded peanuts to make a traditional dish named *Tshidzimba*	a) Shoots, leaves, and unripe fruits: cooked as a side dish; b) Seeds consumed like other legumes [7].	NCU0183	0.065	0.032
Flame Thorn, Flame Acacia (*Senegalia alemquerensis* (Huber) Seigler & Ebinger.)	Muluwa	Shr	N	W	HG			Firewood, crafts	a) Sapling stems: split into strips and used to make weaving baskets named *mufaro which are used to present food for rituals and to serve food for the Khosi*^*2*^; b) Wood: firewood	a) Roots: Aphrodisiac; b) Flexible saplings: decorticated and longitudinally split into thin; band-like strips for weaving baskets; winnowing and storage baskets; c) branches: hedge fencing around cattle enclosures and homesteads; d) Wood: firewood [6]	NCU0027	0.065	0.032
Kiaat, Bloodwood, Paddle-Wood, Sealing-Wax Tree, Transvaal Teak (*Pterocarpus angolensis* DC)	Mutondo	T	N	W	CL			Domestic utensils, medicine	a) Wood: carving household materials such as dishes; desks and tables; b) *Roots: treating sores on the skin*^1^*.*	a) Bark: acceleration of blood formation; heavy menstruation; miscarriage; childbirth; piles; menorrhagia; venereal disease; gonorrhoea; haematuria, bilharzia; b) Wood: carving for doors; door frames; spoons; tool handles furniture and other decorative objects; c) Roots: amenorrhoea; headache; venereal diseases; piles; amenorrhoea; haematuria; bilharzias; treat pulsating anterior fontanelle in babies; d) Fruit: whooping cough [6, 9, 55].	NCU0039	0.065	0.032
Lowveld Bauhinia (*Bauhinia galpinii* N.E.Br)	Mutswiriri	Shr	N	C	HG			Food	a) Roots: eaten as food with soft porridge for young babies b) *Fruits: edible fruit*^*1*^.	a) Roots: used with an infusion of other medicines to make a soft porridge named *tshiunza* for young babies as their main staple food; diarrhoea; enhanced sexual performance; stomach worms; stomach pain; infertility; b) Saplings: wattles in construction of roofs and courtyard walls; Bark and root: stomach spasm [6, 9, 11].	NCU0001	0.032	0.032
Monkey Pod, Eared Senna (*Senna petersiana* (Bolle) Lock)	Munembenembe	T	N	W	HG, F/W			Food, Medicine	a) Seeds pods: edible and eaten during times of drought or hunger; b) Roots: toothache.	a) Pods: eaten but not very palatable and picked during times of hunger of food shortage; b) Roots: mouthwash and toothache; gonorrhoea; syphilis; stomach ache; sterility and barrenness; dysmenorrhoea; or syncope; epilepsy; asthma; toothache [6, 9, 11].	NCU0137, NCU0169	0.065	0.097
Weeping Wattle, African Black Wattle, African Blackwood (*Peltophorum africanum* Sond)	Musese	T	N	C/W	HG		Y	Firewood, medicine	a) *Bark: ulcers on the body*; *sore throats*^2^; *Wood: firewood*^1^; c) The species is found close to rivers**.**	a) Bark: anthelmintic; stomach troubles; colds; coughs; chest complaints; eye sicknesses; rash of the tongue in small children; b) Root and bark: intestinal parasites; tuberculosis; c) Caterpillars on the plant are fried and eaten or stored for future use; d) Leaves: used to cover the body during rituals; e) Roots: sores, ulcers, and blisters of the oral cavity; sore throats; venereal disease; f) Entire plant: menorrhagia, [6, 9, 55].	NCU0006, 16, 43, 141, 160	0.065	0.161
Gentianaceae													
Big Leaf, Cabbage Tree, Fever Tree, Forest Big-Leaf, Tobacco Tree (*Anthocleista grandiflora* Gilg)	Mueneene	T	N	C/W	HG		Y	Medicine, cultural	a) Bark: high blood pressure; b) Leaves: used to cover maize grains to encourage germination when malt is prepared; used to cover female bodies during rituals; c) Important for storing water close to rivers.	a) Bark: malaria; diarrhoea; diabetes; high blood pressure; venereal disease; b) Stamped bark soaked in water with seeds; especially cereal grains; to make the grains produce abundantly when sown; c) Leaves: used to cover millet grains to encourage germination when malt is prepared; worn to cover bodies during rituals; nutrition for cattle d) Important water tree [6, 9, 16].	NCU0044	0.065	0.032
Lauraceae													
Avocado Tree (*Persea americana* Mill.)	Makatapiere	T	E (South Central Mexico)	C	HG	C/N	Y	Food, commercial, medicine, shade, firewood, cultural	**a)** Fruits: edible fruits; trees are grown in small-scale orchards and sold commercially; b) *Leaves and stem: stripped; ground and mixed to make a snuff as well as type of bicarbonate of soda for seasoning vegetables*^1^*; ground and mixed with Amaranthus hybridus to be used as a snuff ingredient*^*1*^*; c) used to treat diarrhoea*^*1*^*; d) Wood: firewood*^*1*^*.*	No known records.	NCU0064, 98, 146, 162	0.194	0.129
Malvaceae													
Bladder Hibiscus (*Hibiscus Trionum* L)	Mandande	H	E (Europe)	W	HG	C/N		Food	a) Leaves: edible vegetable; eaten with porridge and mixed with other vegetables.	a) Leaves: edible vegetables and cooked with porridge [6, 7].	NCU0129, 50	0.032	0.032
Cross-berry, Four Corners, Four-Corners (*Grewia occidentalis* L)	Mulembu	T	N	W	HG			Food	a) Leaves: edible vegetable and eaten with porridge.	a) Leaves: edible vegetable and eaten with porridge; b) Roots: syphilis; venereal disease; bladder ailments [6, 56].	NCU0028	0.032	0.032
Meliaceae													
Cape Ash, Dogplum (*Ekebergia capensis* Sparrm)	Mutobvuma	T	N	W	HG		Y	Firewood, construction, shade	*a*) *Wood: carving to make drums*^1^*; firewood*^1^*;* b) Bark: headaches.	a) Bark: headaches; emetic; heartburn; chest complaints; b) shade and beauty [6, 10].	NCU0011, NCU0052	0.032	0.032
Thunder Tree, Forest Mahogany, Forest Natal Mahogany, Cape Mahogany (*Trichilia dregeana* Sond)	Mutuhu	T	N	C/W	HG		Y	Medicine	a) *Bark: STIs such as gonorrhoea and syphilis*^*1*^*; b) The tree is found in the Chief’s palace and used to guard against bad spirits*^1^*.*	a) Fruits: cooked with vegetables as a condiment; eaten with milk; b) Fruits and seed: cooking oil; polishing women’s leather clothes; polish furniture; c) Bark: used as an enema for general cleaning; kidney troubles which cause impotence; d) ornamental; e) Buried close to graveyards to counter erosion when graves are buried [9, 14, 17].	NCU0008	0.032	0.032
Menispermaceae													
Kidney Leaf (*Cissampelos torulosa* E.Mey. ex Harv. & Sond)	Lukandululo	C	N	W	HG			Medicine	a) *Leaves and stem: flu*^*1*^*.*	a) Leaves and stem: sthroats; dysentery; diarrhoea; spiritual cleansing; b) Leaves: edible vegetable cooked with other vegetables [6].	NCU0030	0.032	0.032
Moraceae													
Cape Fig, Broom Cluster Fig, Bush Fig, Cape Wild Fig, Fire Sticks (*Ficus sur* Forssk)	Muhuyu	T	N	C/W	HG			Food, shade	a) *Planted at the Chief’s palace for shade*^*1*^; b) Fruits: edible fruits are eaten fresh or dried.	a) Fruit: tuberculosis; b) Root: diarrhoea [6].	NCU0009, 34, 166	0.065	0.097
Common Wild Fig (*Ficus thonningii* Blume)	Muumo	T	N	W	HG		Y	Shade, food	a) *Found in the Chief’s palace for shade*^*1*^; found at the foot of a mountain**;** b) Fruits: figs are also eaten when ripe.	a) Fruits: figs are edible when ripe; beverage; b) Latex: used for birdlime; c) semi-parasitic plant growing on the tree is used to treat insanity [6, 13].	NCU0056	0.065	0.032
Red-Leaved Rock Fig, Rock Fig (*Ficus ingens* (Miq.) Miq.)	Tshikululu	T	N	W	HG			Food	a) Fruits: figs are eaten when ripe by humans and animals.	a) Fruits: eaten when ripe but preferred by primates; contains analgesic compounds [6, 10].	NCU0195	0.032	0.032
Myrtaceae													
Waterberry Tree (*Syzygium cordatum* Hoschst.ex C.Krauss)	Mutu	T	N	C/W	HG		Y	Firewood, medicine, shade, drink	a) Found in wetlands and stores water; b) Fruits: beverage; c) Leaves and roots: aids stomach digestion; *d) Bark: sore throats*^*1*^; e) Fruits: eaten when ripe; f) *Wood: firewood*^*1*^*.*	a) Fruit: eaten when ripe; b) Leaves: treating for stomach aches; colds and fevers; c) Leaves and bark: diarrhoea; wounds; d) Roots: headache, amenorrhoea [6, 9, 56].	NCU0045, 58, 88, 159, 161	0.129	0.161
Woodland Waterberry, Waterpear (*Syzygium guineense* (Willd.) DC)	Mutawi	T	N	W	CL			Food	a) Fruits: ripened fruits; b) Found in forests and mountains.	a) Fruits: edible fruits are used by young people [6].	NCU0041	0.032	0.032
Red Guava (*Psidium guajava* L*.*)	Mugwavha	T	E (Central and South America)	C/W	HG	I		Domestic utensils, food, drink, medicine	a) Fruits: beverage; jelly; b) Leaves: stop bleeding wounds; c) *Stem: brooms*^*1*^	a) Fruit: food; b) Whole plant: shade tree; c) Roots, leaves, bark: wounds; venereal disease [9, 14, 15].	NCU0117, 135, 32, 66, 110, 149, 167	0.129	0.161
Ochnaceae													
Yellow-peeling tree (*Brackenridgea zanguebarica* Oliv)	Mutavhatsindi	T	N	W	F/W			Medicine	a) Roots, stem, bark and leaves: medicine to protect homesteads and territories from enemies; b) *Bark: Added to other medicines to enhance its potency*^*1*^.	a) Roots: wounds; swollen ankles; amenorrhoea, worms; mental illness b) Roots, stem, bark, and leaves: used magically to protect homesteads and territories; c) The species discourage opponents in sporting events; offers protection against witchcraft; protects people [6, 9, 10, 57, 58].	Mentioned in survey but species specimen not collected.	0.032	0.032
Olacaceae													
Blue Sour Plum, Tallow Wood (*Ximenia americana* L. var. microphylla Welw. ex Oliv)	Mutuanzwa	T	N	W	HG			Medicine, food	a) Fruits: edible when ripe; b) Bark or powder of the root bark is used to treat diarrhoea.	a) Fruits: eaten when ripe; beverage; b) Bark: remedy for dysentery in children; diarrhoea and febrifuge in adults; c) Semi parasite or epiphyte associated with this plant; used to attract people who do want to return home from their places of work far away; d) Roots: menorrhagia, infertility; venereal disease, headache due to indigestion, blood in faeces, cough, eye diseases [6, 9, 13].	NCU0089	0.065	0.032
Oxalidaceae													
Fishtail Sorrel, Transvaal Sorrel (*Oxalis semiloba* Sond)	Mukulungwane	H	N	W	HG		Y	Food	a) Leaves: chewed to remove a foul taste in the mouth.	a) Leaves: chewed by a person suffering from a tart or sour feeling in the mouth; usually after eating unripe fruit; b) Whole plant or leaves: treatment of haemorrhoids; eye frops [6, 11].	NCU0101	0.065	0.032
Pedaliaceae													
Devil’s Thorn (*Dicerocaryum eriocarpum* (Decne) Abels)	Museto	H	N	W	HG			Medicine	a) *Thorn: rub along the gums to encourage teeth to develop in young children*^1^*.*	a) Leaves: expulsion of placenta and easy delivery; b) Leaves and stem: soap substitute; quicken the expulsion of hanging placenta in cattle and humans; important medicine for a blood disease in cattle known as *mali* (black quarter evil) [6, 9, 10, 12].	NCU0188	0.032	0.032
Phyllanthaceae													
Coastal Goldenlead (*Bridelia micrantha* (Hochst.) Baill)	Munzere	T	N	C/W	HG		Y	Food, medicine	a) Fruits: edible fruits are eaten and are black in colour; b) Found close to rivers and cultivated fields; c) *Bark: bolsters the immune system*^2^.	a) Leaves: eaten when ripe; b) Bark: burns; gonorrhoea; venereal disease; infected wounds; toothache; abortion; c) Long straight branches are laid across the rivers to make bridges; building huts; d) Roots and bark: stomach aches; tapeworms [6, 9, 55].	NCU0004, 15, 165	0.065	0.097
Phytolaccaceae													
Forest InkBerry (*Phytolacca octandra* L)	Thebe	H	E (North America)	W	HG			Food, commercial	a) Leaves and roots: cooked together and eaten with porridge; b) *Leaves: eaten dry to make a type of biltong to be eaten during the winter months*^*2*^*; also during times of drought and hunger*^*2*^*; species is commercialised and sold in informal markets*^*2*^*.*	a) Leaves: cooked and eaten with porridge; spice; b) Leaves and shoots: dried; burnt and mixed with a snuff to serve as a stimulant as well as to give flavour [6].	NCU0017, 37, 48, 127, 132, 145, 153	0.065	0.194
Poaceae													
Sorghum (*Sorghum bicolor* (L.) Moench)	Nkhwe	G	E (Asia)	C	HG			Food, cultural	a) *Seeds: ground into a powder and used in a ritual as an offering to the ancestors in a ‘biting ritual’ named u luma indicating the season for ripening of the first vegetables or fruits*^*1*^**.**	a) Stem: cultivated for their sweet stems [59].	NCU0096	0.065	0.032
Polygonaceae													
Starstalk (*Oxygonum dregeanum* Meisn.)	Muthanyi	H	N	W	HG			Food	a) Leaves: edible vegetable eaten with soft porridge.	a) Leaves: edible vegetable; spice; b) Leaves and shoots: dried; burnt; mixed with snuff to serve as a stimulant and to give flavour [6]	NCU0192	0.032	0.032
Proteaceae													
Broad-Leaved Boekenhout (*Faurea saligna* Harv)	Mutango	T	N	C	HG			Household utensils, crafts	a) Wood: used to construct household utensils and crafts	a) Leaves: used to treat *divhu* or *devhu*; an illness suffered by a man who had sexual intercourse with a woman after an abortion or miscarriage; b) Bark: vagina ulcers; used to wood: workable and durable; c) Roots and bark: venereal disease; bilharzia; d) Roots: cough [6, 9, 33].	NCU0083	0.065	0.032
Macademia Tree (*Macadamia ternifolia* F.Muell)	Mutevu	T	E (Australia)	C	CL			Food	a) *Nuts: ground into a powder and eaten with vegetables and porridge*^*1*^*.*	No known records		0.032	0.032
Rhamnaceae													
False Buffalo Thorn, River Jujube (*Ziziphus rivularis* Codd)	Mulalantsa	T	N	C/W	HG			Firewood, Food, Medicine	a) *Bark: harvested from the eastern and western sides of the plant*^*1*^*; ground and used to treat sores on the skin*^*1*^*; b) Fruits: eaten fresh or dried and sometimes eaten with porridge*^*1*^*; c) Wood: firewood*^*1*^*.*	No known records	NCU0122	0.097	0.065
Rubiaceae													
Rock-Alder (*Afrocanthium mundianum* (Cham. & Schltdl.) Lantz)	Mutomboti	T	N	W	HG			Food	a) Fruits: edible fruit is known as *thomboti*.	a) Fruits: edible fruits; b) Leaves: remedy for illness known as *divhu* (a disease caused by sexual intercourse with a woman who has had an abortion or miscarriage) [17].	NCU0025	0.032	0.032
Strawberry Bush, Quinine Berry, Far Far Tree (*Cephalanthus natalensis* Oliv)	Murondo	T	N	W	HG			Food	a) Fruits: ripened fruit is eaten.	a) Fruits: eaten when ripe; b) Leaves: eye problems in cattle [6, 10, 12].	NCU0172	0.032	0.032
Wild Medlar (*Vangueria infausta* Burch. subsp. infausta)	Muzwilu	T	N	W	HG			Food, medicine	a) Fruits: eaten when ripe or dried; b) Stem: short sticks are crafted from the stem and nailed to the fence of the yard and are thought to protect the homestead.	a) Fruits: eaten fresh or dried; also enjoyed with milk when soaked in water; b) Roots and bark: enhance fertility in women; c) Sticks: nailed all around the fence of a yard to protect the homestead; d) Roots: ulcers in the oral cavity [6, 9].	NCU0031, 35	0.065	0.065
Wild Oleander, African Teak (*Breonadia salicina* (Vahl) Hepper & J.R.I. Wood)	Mutulume	T	N	C/W	HG		Y	Cultural	a) *Tree used to store water*^*1*^*;* b) *Vhavenda proverb: when you are chased by a lion who catches your foot; you pull away; it does not come out; like the root of the mutulume*^*1*^*.*	a) Roots: tachycardia [9]	NCU0010	0.032	0.032
Rutaceae													
Adelaide Spice Tree, Small Knobwood (*Zanthoxylum capense* (Thunb.) Harv)	Munungu	T	N	C/W	HG		Y	Medicine	a) Bark: ground and licked to treat common colds and flu.	a) Roots and stem bark: sore throats; chest complaints; boils; pimples and blood poisoning [6].	NCU0055	0.032	0.032
Lemon Tree (*Citrus limon* (L.) Osbeck)	Tshikavhavhe	T	E (Asia)	C	HG	C/N		Food	a) *Leaves: used to make a tea which can be drunk and used as a medicine for stomach ache and menstrual pain*^*1*^*.*	a) Roots: Venereal disease [6, 9].	NCU0074	0.032	0.032
Sapindaceae													
Litchi (*Litchi chinensis* Sonn)	Nombelo	T	E (Asia)	C	HG			Food, drink	a) *Fruits: eaten during times of hunger*^*2*^; used to make a juice and alcoholic beverage	No known records	NCU0072, NCU0163	0.065	0.065
Sapotaceae													
Red Milkwood (*Mimusops zeyheri* Sond)	Mububulu	T	N	W	HG		Y	Food, drink	Fruits: eaten when ripe and sometimes soaked in milk to make a milkshake.	a) Fruits: edible when ripe; can be soaked in milk or water to make a beverage; dried and stored for future use; b) Root and stem bark: abdominal complaints [6, 13].	NCU0033	0.065	0.032
Wild Plum, Transvaal Milk Plum (*Englerophytum magalismontanum* (Sond.) T.D Penn)	Munombelo	T	N	W	HG, CL		Y	Firewood, domestic utensils, commercial, food, medicine	a) *Wood: cooking spoons; firewood*^*1*^*;* b) Fruits: eaten when ripe; good for nutrition for children; beverage from the fruits used to relieve constipation; c) Species is found near wetlands.	a) Fruits: edible; juice; fermented beverage; b) Roots: remedy for abdominal pains; c) A semi parasite or lichen of this plant is used as an ingredient of medicines; prepared and burn to invoke ancestral spirits during malombo (*Vhasenzi*) or mbila (*Vhalemba*) cults [6, 13].	NCU0038, 61, 86	0.161	0.097
Solanaceae													
Black or Common Nightshade (*Solanum nigrum* L)	Muxe	H	E (Europe)	W	HG			Food	a) Leaves: cooked and eaten with porridge and other vegetables.	a) Leaves: cooked and eaten with porridge; with meat or other vegetables; malaria and dysentery; anal are a known analgesic effects on toothache; b) used as a cholagogue; c) Roots and leaves: wounds [6, 7, 15].	NCU0108	0.032	0.032
Ulmaceae													
White Stinkwood (*Celtis africana* Burm. f)	Mumvumvu	T	N	C	HG		Y	Medicine	a) Stem of branches: used to make magical sticks which are driven into the ground to protect against witchcraft.	a) Bark: magical properties; nose and ear drops; toothache; b) Branches: used to protect the homestead [6, 9].	NCU0106	0.032	0.032
Urticaceae													
Fever Tea, Lemon Bush (*Lippia javanica* (Burm.f.) Spreng))	Musudzungwane	Shr	N	W	HG			Domestic utensils, medicine	a) Leaves: used to make a tea which is used to treat common colds and to boost immunity; *leaves are crushed and sniffed to treat nose bleeds and used as appendages to block nose bleeds*^*1*^; mosquito repellent; malaria; *b) Stems: brooms*^*1*^*.*	a) Leaves: coughs; flu and headaches; general body sickness; malaria; dysentery; diarrhoea; anthelmintic; asthma; tick toxicant; b) Roots: burnt and pounded to produce a medicine that is applied cuts and sprained joints; dislocated joints [6, 9, 12, 13, 60]	NCU0093, 124	0.065	0.065
Mountain Nettle (*Obetia tenax* Friis)	Muvhazwi	H	N	C/W	HG, CL			Food, medicine	a) Leaves: cooked and eaten with porridge as a nutritious meal; b) Stem: used to treat snake bite wounds.	a) Leaves: cooked and eaten with porridge; b) An epiphyte or semi parasite growing on the plant is used for treating snake bite; c) Bark: source of fibre cordage; ox-whips; mats; thatching; game traps; and sieves for straining beer [6, 7].	NCU0097, NCU0143	0.065	0.097
Stinging Nettle (*Urtica dioica* L)	Dzaluma	H	E (Europe, Asia, Western, Northern Africa)	W	HG			Food	a) Leaves cooked and seasoned with salt; fresh tomatoes are added to make the relish which is then eaten with porridge	a) Leaves: cooked and eaten with porridge [7].	NCU0018, 23, 50, 75, 128	0.065	0.161
Verbenaceae													
Bird’s Brandy, Bird’s Beer (*Lantana rugosa* Thunb)	Tshidzimbambule	Shr	N	W	HG			Food	a) Fruits: the purple berries are eaten when ripe.	a) Fruits: eaten when ripe; b) Leaves and stem: treat troublesome eyes; c) Leaves: bronchial infections; abdominal complaints; anti-emetic; eye injuries d) Roots: fever [6, 9, 11].	NCU0036, NCU0173	0.032	0.065
Viteceae													
Wild Grapes (*Rhoicissus tomentosa* (Lam.) Wild & R.B.Drumm)	Ndirivhe	C	E (Europe)	C	HG			Food	a) Fruits: consumed for food.	a) Fruits: preferred by monkeys but is also eaten by people in Vhavenda; usually eaten out of hunger because it is not very palatable and has a sickly sweet taste; quenches thirst when eaten [6]	NCU0091	0.032	0.032

Family/scientific name/local name; Habit (*C* climber, *G* grass, *H* herbaceous, *Shr* shrub; *T* tree); *BO* biogeographic origin (*N* native, *E* exotic); Status (*W* wild, *C* cultivated, *C/W* cultivated and wild); *CS* collection sites (*HG* home garden, *CL* cultivated land, *F/W* evergreen forest/deciduous woodland); *AP* alien plants (*I* invasive, *C/N* casual/naturalised); *P* propagation in home gardens (*Y* yes, *N* no); Use value; Description (entirely new plant use records are indicated in italics by the superscript ‘1’ and partially new records of plant uses are indicated in italics by superscript ‘2’); Literature on Vhavenda ethnobotanical use; Voucher numbers; UVI (Use Value Index); RFI (Relative Frequency Index)

### Species origin, status and habitat

The majority of plants were native species (73.8%) compared to exotics (26.2%). The average year of settlement in the region was 60.5 years suggesting a considerable period of knowledge assimilation and experimentation with the local flora. The habits of the species were trees (53.2%), herbs (21.3%), shrubs (8.5%), climbers (5.3%) and grasses (1.0%). The communities of Tshidzivhe, Vuvha, Lwamondo and Tshiendeulu were surrounded by small patches of montane forests and deciduous woodlands in river valleys and higher elevation slopes. Plant samples were collected for identification from home gardens (89.7%), cultivating fields (6.9%) and montane forests and deciduous woodlands (3.4%).

**Fig. 2:**
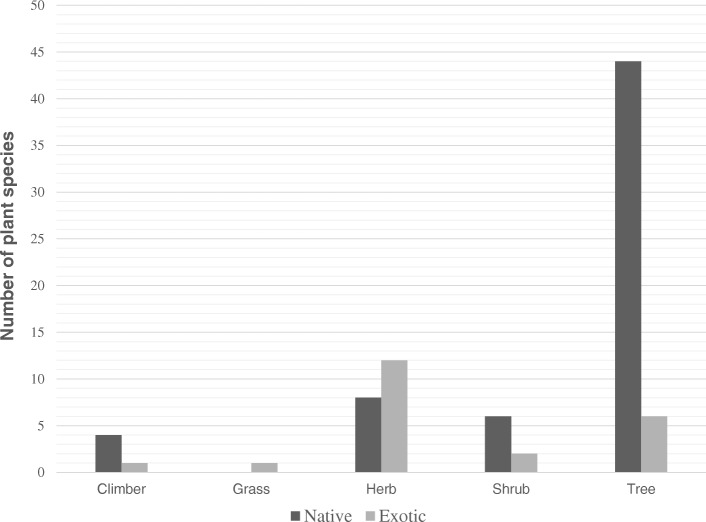
Number of exotic and native plant species separated by habit in the Vhavenda community, Limpopo Province, South Africa

The majority of plant species used were wild (54.8%) compared to cultivated species (20.2%) whilst 25% were both harvested in the wild and cultivated. When the plants were separated by habit, native species were mainly trees, and exotic species were mainly a combination of herbs and trees (Fig. 2).

**Fig. 3:**
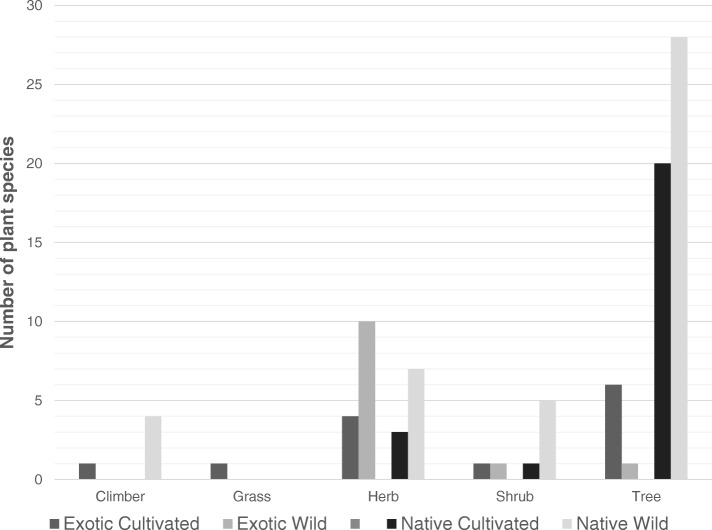
Number of exotic and native cultivated and wild species separated by habit in the Vhavenda community, Limpopo Province, South Africa

Considering only the exotic cultivated species (Fig. 3), these were mainly trees and herbs that have originated from areas of Europe, North and Central America, Asia, Australasia and Tropical Africa before their introduction to South Africa (Table 1). Of the exotic tree species, *Carica papaya* L, *Citrus limon* (L.) Burm. F and *Persea americana* Mill were reported as naturalised or casuals in South Africa (Table 1).

Of the exotic cultivated herb species documented, *Psidium guajava* L and *Ricinus communis* L were listed as invasive. Exotic wild species were mainly herbs (Fig. 3) that were of European, North or Central America, North African and Asian origin. Of the exotic herbs species listed, *Amaranthus hybridus* L, *Bidens pilosa* L, *Chenopodium album* L, *Colocasia esculenta* (L.) Schott, *Hibiscus trionum* L, *Obetia tenax* Friis and *Sonchus oleraceus* (L.) L were reported as naturalised or casuals in South Africa (Table 1).

Native cultivated species were mainly trees (Fig. 3) to name a few included *Celtis africana*, *Combretum erythrophyllum* (Burch.) Sond, *Faidherbia albida* (Delile) A. Chev, *Faurea saligna* Harv, *Philenoptera violacea* (Klotzsch) Schrire and *Rauvolfia caffra* Sond. The Vhavenda continue to exploit native wild species mainly trees (Fig. 3), for example, *Afrocanthium mundianum* (Cham. & Schltdl.) Lantz, *Annona senegalensis* Pers, *Carissa edulis* (Forssk.) Vahl, *Cephalanthus natalensis* Oliv, *Combretum collinum* Fresen, *Diospyros lycioides* Desf, *Englerophytum magalismontanum* and *Ekebergia capensis* Sparrm.

### Species use and frequency

To our knowledge, six of the species documented have not been recorded in the ethnobotanical literature of local plants used by the Vhavenda, prior to this study (Table 1). Three of these species are exotic including *Litchi chinensis*, *Macadamia ternifolia* and *Persea Americana*, and other species include the exotic *Athyrium filix-femina* (L.) Roth and the indigenous *Croton sylvaticus* Hochst and *Ziziphus rivularis* Codd. The local uses for these species appear to remain unpublished, as similar Vhavenda ethnobotanical studies documenting the use of these plants could not be found in the literature. New Vhavenda traditional use records for 68 species were also identified as well as 14 partially new plant uses (i.e. variations on previously recorded uses) and presented in Table 1.

**Fig. 4:**
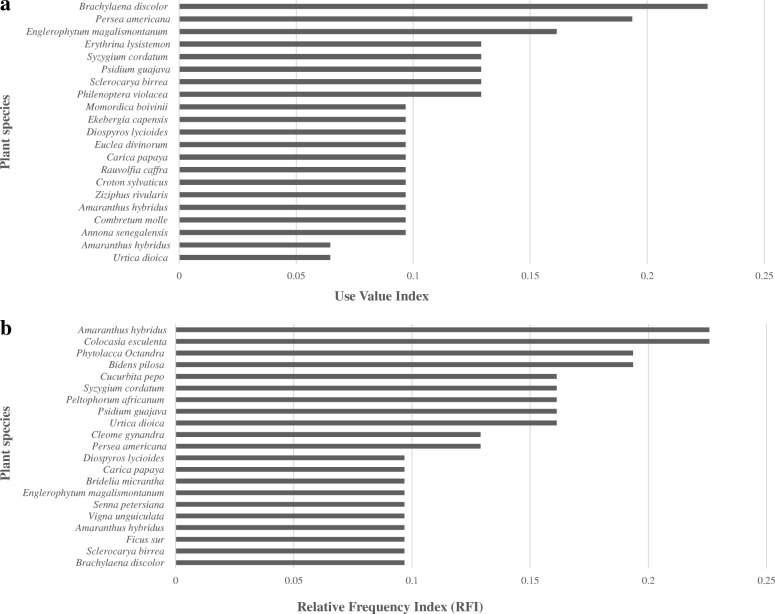
**a** Use Value Index (UVI) and (**b**) Relative Frequency Index (RFI) of plants citied by the Vhavenda, Limpopo Province, South Africa

The species with the highest use value index were recorded for the *Brachylaena discolor* DC (0.23), *Englerophytum magalismontanum* (0.16) and *Persea americana* (0.19) representing the highest number of plant uses of all species documented (Fig. 4). The species with the highest relative frequency included *Amaranthus hybridus* (0.23) and *Colocasia esculenta* (0.23) as the most frequently cited species by informants (Fig. 4b).

Of the species identified, the majority were used for food (36.0%), medicine (26.1%), firewood (11.8%), shade (5.0%), cultural purposes (5.0%), drink (4.3%), domestic utensils (4.3%), commercial use (2.5%), charcoal (1.9%), construction (1.2%), crafts (0.6%) and as indicator species for soil fertility (0.6%). Of the 84 species identified, 61.7% were multi-use. Of all species identified, the leaves (25.7%), fruits (20.8%), wood (16.0%), bark (13.9%), roots (9.7%), stem (8.3%), seed (2.8%), thorns (0.7%), latex (0.7%), flowers (0.7%) and the whole plant (0.7%) were used.

### Cultural practices associated with Vhavenda plants

The Vhavenda integrate a range of strategies for protecting plant resources including prohibitions against certain plants from being used, the promotion of sustainable harvesting practices and the propagation of plant species to support ecological restoration (Table 2). The most common strategy for managing plant resources included taboos preventing trees from being cut down, used as firewood or taken back to the homestead for 11.9% of species. The following trees were prohibited from being cut down: *Anthocleista grandiflora* Gilg, *Breonadia salicina* (Vahl) Hepper & J.R.I. Wood, *Bridelia micrantha* (Hochst.) Baill, *Combretum molle* R.Br. ex G.Don, *Ekebergia capensis*, *Englerophytum magalismontanum*, *Ficus sur Forssk*, *Mundulea sericea* (Willd.) A. Chev, *Pterocarpus angolensis* DC and *Sclerocarya birrea* (A.Rich.) Hochst. The tree *Mundulea sericea* is prohibited from being used as firewood in homesteads where cattle are present, and *Celtis africana* cannot be planted in the homestead. *Sclerocarya birrea* fruits can only be harvested when they have fallen to the ground; otherwise, the culprits were believed to experience a fever or snakes would appear in their homesteads. *Brackenridgea zanguebarica* Oliv is prohibited from entering Vhavenda homesteads, and a failure to adhere to the taboo results in sterility among household members. *Brackenridgea zanguebarica* is also prohibited from being used for firewood, hedge fencing, building work or wood carving.

**Table 2:** Traditional practices for managing plant resources in targeted villages

Traditional practices	Duthuni	Tshidzivhe	Vuvha	Lwamondo	Mashau	Tshiendeulu
Consultation of the *Khosi* or *Mukoma* to seek permission before harvesting plants		X		X	X	X
Penalty for cutting down trees and the payment of a fine of ZAR 1000 (75USD)		X				
Stem bark is harvested on the eastern side of the plant	X					X
Only the lateral roots of plants are collected	X		X	X	X	X
Soil is covered back over harvested roots	X					X
Propagation of plant species in home gardens	X	X	X			
Taboos preventing trees from being cut down, used as firewood or being placed in the homestead	X	X	X	X	X	X
Edible fruits from trees are only harvested when the fruits have fallen to the ground or ripened		X				X
Some species are only harvested for their tender leaves		X				X
Only the deadwood if trees is collected for firewood			X	X	X	
Consultation with the ancestors before harvesting a plant			X			
Women are not allowed to collect plants during menstruation			X			X
Plants harvested only from specific areas				X	X	
Prohibitions from harvesting plants from the same area each year				X	X	
Some species are only planted and harvested during certain seasons following ritual ceremonies by *Makhadzi* or traditional leaders.		X				X
Species only harvested when the flowers are present indicating the maturity of the plant						X

Legend: X indicates the presence of traditional practices for managing plants in a village

The Vhavenda have developed selective harvesting practices, for example, plants are taken from specific areas and it is prohibited from returning to the same area in a year allowing the plants to recover. Only the tender leaves of *Amaranthus hybridus*, *Cucurbita pepo*, *Momordica balsamina* L, *Momordica boivinii Baill* and *Phytolacca octandra* L are selected allowing the leaves to reach maturity. *Cucurbita pepo* is only harvested after the flowering of the plant, indicating the time for harvesting when the vegetable has reached maturity. The leaves of *Cucurbita pepo* and *Momordica boivinii* must not be harvested or touched by menstruating women; otherwise, it is believed that the vegetable will shrink in size. The wood of *Pterocarpus angolensis* is often placed in home gardens to prevent menstruating women from entering the garden. Similar beliefs are also extended to the harvesting of fruits for *Sclerocarya birrea* where only elder women who have experienced menopause are permitted to harvest the fruits. The bark from trees such as *Ziziphus rivularis* is harvested from the eastern side of the tree, due to a belief that bark harvested from this side has the highest nutritional level of all sides and the wound is covered with a mixture of soil and water to aid the recovery of the tree. Many of the practices described traditional healers to harvest plants for medicines were conducted in a manner to prevent the destruction of an entire tree, for example, only the horizontal edges of tap roots were removed from each side of the plant and the remaining roots covered back over with soil.

Of the 84 plant species identified, 34.5% of the species seeds were stored and saved for propagation in home nurseries and were mainly native (82.8%) compared to exotic (17.2%) species (Table 1). This practice is motivated by the need for individuals to grow important herbs or trees for food or their medicinal value; however, groups of women were also observed developing nurseries in their home gardens for the restoration of indigenous montane forests close to sacred groves and river valleys.

### Cultural institutions associated with Vhavenda plants

In the villages of Tshidzivhe, Lwamondo and Tshiendeulu, the *Khosi* and *Mukoma* continue to play an important role in monitoring compliance of rules to harvest indigenous plant species in an area under his jurisdiction and the reporting of unsustainable harvesting practices. The *Khosi* is also consulted by traditional healers prior to harvesting trips undertaken by those who desire firewood or medicines from the forest. Often, the *Khosi* or *Mukoma* uses this traditional system to monitor plant populations, and if overharvesting or ill behaviour is observed, individuals are called to the *Khosi’s* palace and issued with a sanction in the form of a fine.

The agriculture cycle of the Vhavenda is imbued with a form of wisdom tied the contemporaneous life cycles of different species interlinked with knowledge of their environmental requirements and seasonality, social norms, taboos and ceremonies to indicate rules for knowing when to cultivate, plant and harvest. The timing of agriculture cycles for soil preparation, planting and cultivation are determined by *dzima* which refers to different seasons determined by the phases of the moon. The first *dzima* occurs in September (*khubvumedzi*) or October (*tshimedzi*) where the land is cleared, the soil is tilled and seeds are sown. Public rituals and ceremonies are integrated into the agricultural practices of the Vhavenda in the villages of Tshidzivhe and Tshiendeulu, for example, one informant described the ritual of *u suka* referring to the mixing of seeds which were mixed with a medicine and sown into the perimeters of the field before the ploughing season to enhance soil fertility. Informants reported that seeds were also doctored before planting and rain-making rituals were conducted to enhance the crop yield.

In January (*phando*), the second *dzima* commences in summer (*tshilimo*) where vegetables and crops are harvested, and a second round of planting commences. Other rituals were performed to give thanks to the ancestors after the harvest, for example, January was often indicative of the ripening of wild fruits and vegetables which were consumed after the first fruit rituals named u *tungula* or *u lumisa* were performed as thanks to the ancestral spirits. When the crops are ready for harvesting, the *Makhadzi* picks a sample of each vegetable or fruit that are cooked with herbs. She performs a ritual where a libation of unfermented beer is poured into clay pots as an offering to ancestral spirits and to inform that cultivation is ready; this is named *u luma* the biting of the first fruit. The fruits of the Marula Tree (*Sclerocarya birrea*) are often harvested during this time when the fruits have fallen to the ground indicating the time for harvesting. A third *dzima* commences from May (*shundunthule*) onwards during the winter period (*mavhuyahaya*) where the second round of crops are harvested, and a rest period takes place until September where the fields are left fallow and domestic livestock graze on the remains of the harvested crops. Traditionally, this period was also marked by ritual ceremony in July named *thevhula* a form of thanks giving to share the final harvests with the ancestral spirits.

### Knowledge acquisition and barriers for knowledge transmission

Plant knowledge and management practices were reported to be transmitted from relatives (grandparents and parents) (48.4%), self-taught through time spent planting and harvesting plants on the land (19.4%), through apprenticeships with traditional healers (16.1%), initiation schools (9.7%) and clan gatherings (6.4%). Acquired plant knowledge from everyday interactions was taught through relatives by spending time accompanying family on plant harvesting trips, planting and collecting plants for food and during meal preparation. Some individuals also specified that songs and fables were taught by family members to illustrate of forms of appropriate conduct and were often imbued with lessons in ecology and proper ways of respecting one another. Others taught themselves how to identify plants through time spent planting and harvesting or sought the guidance of more knowledgeable individuals in the village. Traditional healers learnt about plant medicines through apprenticeships with more experienced healers. Formalised cultural institutions such as initiation schools where girls and boys learn about the laws of life, the environment, sexuality, gender responsibilities and respect for the elderly also play a role in transmitting plant-based knowledge. Others claimed plant knowledge was learnt during clan gatherings and ritual ceremonies. Interviews with elders identified that taboos and belief systems were considered important for encouraging individuals and communities to follow traditional customs and laws, for example, clan gatherings would occur at the *Khosi’s* palace where individuals were reminded how to observe local taboos, rituals and practices. In turn, traditional leaders play a prominent role in reviving rituals to set an example for others during communal celebrations and to act as role models for the rest of the community.

Interviews with elders revealed a disjuncture between knowledge exchange between elders and youth and a loosening of more formalised learning platforms for knowledge sharing about plants, taboos and social norms. Initiation schools, clan gatherings and communal ceremonies such as the vegetable and first fruits ceremonies are infrequent and only observed in villages that are isolated and maintain their traditions. The interchanges between knowledge holders, the place where knowledge is shared and the novice who is able and willing to experience such memories are few. There are fewer skilled experts ‘on the land’ by local people spending more time in the classroom at school or university, in the town centre, or conducting business away from home. Traditional leadership roles of the *Khosi* and *Mukoma* have changed with traditional leaders spending less time in their communities and fewer clan gatherings being held in villages resulting in partial knowledge of village affairs and issues on the ground. Traditional healers may no longer consult with the *Khosi* before harvesting and firewood trips. This is observable where people collect plant material in the absence of members of the tribal authority and do not observe the taboos and traditional cultural practices.

Observations by interviewees identified how cultural practices and beliefs are re-evaluated internally by young generations. As education becomes a primary facet of growing up in Venda, school and university education has devalued cultural institutions and traditional systems of authority over land and natural resources. New roles exist in the community for youth who have returned to their homes from university to exercise household decision-making power and challenge the authority and traditions of their elders. The emergence of new ideals among youth and adults associated with acquiring wealth and status has led to a form of economic stratification and lessening of communal traditional values and responsibilities. Informants stated that Christianity was originally initiated through mission education, and their teachings have influenced perceptions of practices and beliefs associated with ancestral spirits. The main observable impact is the demeaning of local cultural practices, beliefs surrounding taboos and rituals associated with ancestor veneration challenging the medicinal, magical and religious aspects of Vhavenda life.

## Discussion

### Characterisation of documented plant species

Fabaceae represented the most important plant family with the highest diversity of plant species recorded among the Vhavenda. Similar studies in the region have highlighted Fabaceae as the most important family for medicinal plants [10, 11, 33] and the treatment of livestock diseases [12] whilst others have reported the importance of the Apocynaceae for the use of plants for traditional beverage-making plants [13]. The result is unsurprisingly considering Fabaceae represents the third largest family of angiosperm plants with over 20,856 species documented worldwide [34]. The most frequently recorded plant species found in this study are trees followed by herbs, which is similar to other ethnobotanical studies on the Vhavenda [12, 13, 33]. Trees and herbs have also been recorded as the most dominant life forms used by other cultural groups such as the Bapedi in the Limpopo Province [14]. The preference for trees and herbs among the Vhavenda may reflect the ease of collecting herbs both in the wild, through their cultivation in home gardens and the all-season availability of trees, providing a continuous supply of ecosystem services for local people [35]. The Vhavenda possess a long-term accumulation of learnt knowledge of the use values of biologically diverse indigenous plant species as evidenced through the dominance of mainly native species (73.8%). There were preferred places for plant gatherings although many of the collected species used for vouchering were mainly identified in home gardens and cultivated lands. Although the majority of informants continue to harvest wild plant species from forests and woodlands, the relatively low frequency of these species for vouchering may also relate to the declining presence of forest and woodland cover in the region due to the establishment of pine and eucalyptus plantations and residential housing expansions [36]. Many species documented in this study were wild (54.8%) compared to cultivated species (20.2%). In the Vhembe District, other researchers also documented a dominance of wild plant species used by the Vhavenda because the properties of wild plants are perceived to be more potent compared to cultivated species for medicinal purposes [12]. It is also important to note that some individuals also gathered wild plants along trails between residential areas, cultivated lands, forests and woodlands. Other studies have shown a change in the collection of plants in communal lands to collecting species associated with disturbed sites located closer and within residential areas [37, 38]. The presence of both wild successional and cultivated species highlights the elevated importance of disturbance regimes for plant utilisation and management.

The Vhavenda use a relatively lower number of exotic plants (26.2%) but have managed to domesticate and cultivate a range of exotic trees in home gardens and small-scale orchards, for example, *Macademia ternifolia* and *Persea americana* are in demand within local markets, and as a means of improving yields and incomes for rural farmers. *Carica papaya*, *Citrus limon*, *Litchi chinensis* and *Psidium guajava* are favoured as domestic fruit trees. Exotic cultivated herbs such as *Amaranthus hybridus*, *Colocasia esculenta* and *Cucurbita pepo* are often consumed as vegetables and have become a vital component of the local diet and economy. Two invasive species used by the Vhavenda, *Psidium guajava* and *Ricinus communis*, have also been documented by other researchers as part of Venda’s repository of useful plants [14, 15]. Despite the relatively low utilisation of exotic and invasive species, their use is also indicative of the positive role of widely dispersed species in the process of ethnobotanical adaptation as new species become introduced over time.

To our knowledge, new ethnobotanical literature for six new species not previously documented in the scientific literature are used by the Vhavenda including *Athyrium filix-femina*, *Croton sylvaticus*, *Litchi chinensis*, *Macadamia ternifolia*, *Persea americana* and *Ziziphus rivularis*. In addition, 68 new and 14 partially new uses of Vhavenda plants were also identified for a variety of species providing a more complete insight into the pattern of plant use in the Vhavenda ethnobotanical record. New documented species records and use values indicate that ethnobotanical knowledge of the Vhavenda is incomplete particularly regarding the utilisation of exotic species which is largely overlooked. Plant species are harvested not only for food and medicine but also for sources of firewood, shade, cultural and spiritual purposes, drink domestic utensils, commercial use, charcoal production, construction, crafts and as indicator species for understanding soil fertility. Other studies on the Vhavenda have highlighted similar uses although there has been an inherent bias towards studies of plants for food [7, 8], medicines [9–12] and other cultural and spiritual purposes [16, 17].

The native *Brachylaena discolor* was cited as having the highest number of uses for firewood, construction, crafts, domestic utensils, charcoal, medicine and its cultural use. The wood is favoured for carving spoons, fences, huts and poles as well as roofs, wall posts and tool handles [16]. The wood is also used to provide a support for the neck to correct bad posture in children and to make threads for ties. The leaves are used to make a medicine to treat roundworm [16]. *Persea americana* was cited as having the highest number of uses for food, medicine, shade, firewood and its commercial and cultural value. *Persea americana* are being increasingly cultivated by emerging farmers in community orchards to support local livelihoods. Although tropical fruit production is not traditionally part of Vhavenda culture currently, the Vhembe District represents the second largest district to produce mangoes, avocadoes, macadamia nuts and bananas with the Limpopo Province being the largest exporter of avocadoes in the country [24]. The species most frequently cited by informants included *Amaranthus hybridus* and *Colocasia esculenta*, both exotic to South Africa that have become naturalised in South Africa and are now favoured as vegetables and for their nutritional value. *Amaranthus hybridus* is thought to bolster the immune system and alleviate high blood pressure. The ethnobotanical records for these species demonstrate similar uses, for example, *Amaranthus hybridus* is a popular leafy vegetable, the leaves are eaten with pumpkin leaves and flowers and eaten with *Corchorus tridens* L, the species is also used to test suitability of baby’s type of food during first 3–4 days after birth. If the baby shows symptoms of diarrhoea, it is given a soft porridge named *khongodoli* instead of *ntsu* a liquid food [6–8]. *Colocasia esculenta* has previously been documented as a favoured leafy vegetable among the Vhavenda [7].

### Cultural practices and institutions for plant management and conservation

The current study identifies a range of species-specific taboos associated with ten plant species to prevent them from being cut down, used as firewood and taken inside homesteads. These species are important as they are also favoured for their use as firewood, edible fruits, famine foods and medicines. Other studies have also documented similar taboos preventing the same trees from being cut down, for example, *Anthocleista grandiflora* ‘maintained the status of the territory,’ *Combretum molle* is a favoured shade tree and *Ekebergia capensis* attracts rain [16]. Taboos among the Vhavenda have also been implemented to prohibit certain trees from being misused because of medicinal and magical beliefs associated with them. Sacred trees used to protect territories or homesteads named ‘*u vhea mudi*’ are tabooed, for example, *Philenoptera violacea* is not used for firewood because of a belief that burning it inside the homestead can lead to the dissolution of marriage [6, 16]. Some species have traditionally been used to protect against invasion by other tribes and against natural disasters. Similar species are also tabooed because they are used to protect families inside the homestead, for example, *Celtis africana* is used to protect a family against witchcraft but is not to be planted inside the homestead [16, 39]. The association of taboos with species that have traditionally been protected by the Vhavenda is also well documented, for example, *Sclerocarya birrea* are protected by the Vhavenda for their edible fruits, use as famine foods during times of hardship, medicines and shade [6]. Taboos associated with *Sclerocarya birrea* are enforced through beliefs such that violators or the community experience illnesses or other punishments emanating from their actions [6]. *Sclerocarya birrea* should not be cut down because it is believed to hold the land together and if removed will prohibit the rain from falling [16]. Only male trees of *Sclerocarya birrea* are cut down, and individuals face heavy fines if the tree is felled without the permission of the *Khosi* [39]. Taboos have continued to preserve tree species throughout Africa, for example, among the Bolero in Malawi traditional norms and practices for protecting sacred tree species have resulted in the protection of *Faidherbia albida* for its role in moisture retention and enhancing soil fertility for crop production during drought [40].

The Vhavenda carry out a number of practices that seek to promote sustainable harvesting practices, for example, extraction of the bark from the eastern side of a shrub or tree has also been observed among other Vhavenda researchers [6] and the Bapedi [35] due to a belief that bark harvested on this side of this tree is more potent for medicinal purposes. Other researchers have suggested that this method prevents ring barking of the tree, and because the tree receives sunlight from the eastern and western side of a tree, this supports faster healing [41, 42]. The harvesting of only the tender leaves of selected plant vegetables and flowering of pumpkins indicating the maturity of the vegetable as observed in this study has also been documented among the Mantheding community in the Limpopo Province of South Africa [43]. The following cultural practice can also be associated with life history taboos which are applied when a cultural group bans the use of certain vulnerable stages of a species life history based on its age, sex or reproductive status [44]. In these cases, vegetables are harvested to allow them to reach maturity, bear seed and grow into the next season. The act of refilling soil over harvested roots of medicinal plants among the Vhavenda has also been observed among the Shona of Zimbabwe which is strengthened by a taboo suggesting that a failure to adhere to the practice will result in worsening sickness of the patient [45]. Other researchers have highlighted a number of taboos associated with *Brackenridgea zanguebarica* among the Vhavenda, for example, harvested roots or bark of this tree is prohibited from entering the homestead and women who touch the plant may experience non-stop menstruation [39]. The species is thought to cause health problems for people who harvest the roots or bark without following the correct procedures [39]. Segment taboos occur when a cultural group bans the utilisation of a species for specific time or age, sex or social status [44]. Segment taboos associated with women, children, menstruating females and parents of new-borns are common [44]. The prohibition of women harvesting plants during menstruation is not specific to the Vhavenda but is also encountered among African groups such as the Bapedi who believe that menstruating women will decrease the fertility of food crops, which may originate from cultural beliefs surrounding human health risks [3, 44].

Plant propagation through the cultivation of herbs and climbers and medicinal plants in home gardens has played a role in the conservation of indigenous vegetation. For example, the Kei-Apple (*Dovyalis caffra* (Hook.f. & Harv.) Sim) and Oval Kei Apple (*Dovyalis zeyheri* (Sond.) Warb) still survive in areas of the Soutpansberg that were previously occupied by the Vhavenda [46]. Propagation of indigenous fruits and vegetables through dispersal of seeds in the homesteads for use by family members has also been observed among the Mantheding community in the Limpopo Province of South Africa [43]. However, among the Vhavenda, the motivations for plant propagation have a range of implications for the conservation of wild indigenous plants to support ecological restoration projects in the region. The various strategies employed by the Vhavenda to protect their plant resources provide in situ management methods for sustaining plant resources.

Traditional leaders in the study continue to play an important role in extending authority to monitor compliance of social norms and rules governing the harvesting of indigenous plant species. Similar roles have also been observed in other regions of the Limpopo Province where traditional leaders monitor indigenous plants use in villages through the sanctioning of fines and penalties for non-compliance [3, 43]. Similarly, specialists such as traditional healers and female regents (*Makhadzi*) are significant in supporting institutional memory through the enactment of ritual ceremonies associated with plant utilisation and management. Cultural practices such as the performance of rituals to ancestral spirits through offerings of beer and food to give thanks for the year’s harvests or prior to harvesting trips promote a value system that enforces a respect for all living forms. Traditionally, the *Khosi* or *Mukoma* declare the Marula season open for harvesting and it was often customary to perform a ritual before the Marula is brewed to make *mukumbi*, a beer [16]. Households can then brew their own beer, and samples are taken to the traditional leader for tasting which are cleansed by medicines overseen by the Makhadzi before tasting and then offered to the ancestors [19]. Thanksgiving ceremonies after the harvesting period are also performed, for example, the *Makhadzi* performs the *thevhula* ceremony where unfermented beer is poured into clay pots as an offering to the ancestral spirits [19]. The ceremony is also used to ask for appeasement in terms of other phenomena that have disturbed harmony within the community including disease, bad harvests and natural disasters and to call the rain for the next season’s harvest [19]. Similar cosmological beliefs have also been observed among the Bolero in Malawi where rituals and offerings are made to God and the ancestors for rain. The cooperation of the ancestors is ensured through offerings, particularly of beer and food. When the ancestral spirits are aggrieved, usually through breaching of a moral code (or social contract), anger is believed to manifest in communal hardships through drought; therefore, rituals are held in the village to call upon the ancestors to deliver rain [40].

### Changing dynamics of knowledge transmission in Venda

Plant knowledge is mainly perceived to be transmitted vertically (from parent to offspring) through ‘learning by doing’ from everyday interactions with relatives from helping parents to plant and harvest different species, gathering plants in the forest for food, medicines and firewood as well as preparing plant materials for meals. Other popular forms of knowledge acquisition among the Vhavenda also include oblique transmission through formal apprenticeships with traditional healers, initiation schools and clan gatherings. This follows other studies on knowledge acquisition suggesting that learning stages take place through hands-on experience and observation often reinforced through apprenticeships with knowledgeable elders [47, 48]. From an intergenerational perspective, informants highlight the role of declining traditional platforms for knowledge exchange between elders and youth observed through the lessening of storytelling, initiation ceremonies, clan gatherings, communal rituals and ceremonies. These social mechanisms allow for cultural internalisation and as ‘knowledge carriers’ to enable the remembrance of rules, taboos and practices associated with plant use and management to be inculcated. Traditional cultural practices conducive to biodiversity conservation are no longer practised owing to a lack of responsibility among tribal leaders and community members to encourage the uptake of cultural practices and adherence to traditional rules and regulations as has been reported in other African studies [16, 39]. These cultural institutions play an important role in passing on institutional memory and supporting communal action; however, their decline also undermines the continuation of plant-based knowledge and management practices for utilising and protecting plant resources on communal lands.

Elders also highlight the interplay of changing value systems among the youth through the introduction of western education systems and new religious beliefs that have led to a lack of plant-based knowledge, less time spent on the land and adherence to moral codes, rituals and taboos. Formal education has been associated with ‘less time spent in the bush’ and negatively correlated with indigenous knowledge [47]. In this study, informants identified the schooling system as a causal factor driving changing value systems among younger generations as education eclipses traditional forms of knowledge transmission. Intergenerational frictions materialise through perceptions of local and indigenous knowledge as inferior to knowledge gained through formal education among the youth and observed rebellions against rules of conduct including adherence to taboos [16]. Continuity in the methods of interaction and engagement with plants and the land through the presence of knowledgeable elders and apprentices on the land is intrinsically tied with the continuity of the knowledge itself. Therefore, a lack of elders and youth engaging with the land and plants in daily life disrupts this system of knowledge acquisition. Similarly, changing religious beliefs can create epistemological and intergenerational frictions [40].

Emerging tensions are historically grounded, and our case study identifies a pattern of past colonialism and apartheid that has led to a transformation of traditional leadership structures and the introduction of new belief systems. Firstly, the youth’s lack of consideration for traditional leader’s authority is associated with the belief that traditional leaders complied with the demands of the National Party as a way of holding onto power; therefore, younger generations often associate these structures as entrenched in the old systems of apartheid [19]. Secondly, on the arrival of the Christian missionaries during the colonial era, their teachings aimed to acculturate the Vhavenda by advocating monotheism and depicting ancestral spirits, deities and other gods of the Vhavenda as false [19]. These frictions have demeaned local cultural practices such as consultation with ancestral spirits, traditional healing, thanksgiving rituals and myths and taboos of the Vhavenda [17]. The institution of the role of the *Makhadzi* is also changing, for example, few people will submit themselves to the ritual practices associated with ancestor veneration, thanksgiving and initiation ceremonies and would rather consult with Christian religious leaders for guidance [19]. The impact of these processes is too complex to unravel here but may explain some of the root causes of current tensions and barriers influencing the erosion of traditional forms of knowledge transmission.

## Conclusion

The Vhavenda of South Africa maintain a complex ‘knowledge-practice-belief’ system surrounding the utilisation, management and protection of plant resources. The records of new plant use documented in this study and the elaboration of new uses of well-documented species are useful for providing a more comprehensive insight into the patterns and practices of plant use among the Vhavenda. Their knowledge of plant uses is extensive including a diversity of native, exotic, wild and cultivated species. Knowledge holders cultivate and collect a variety of species in home gardens cultivated lands, montane forests and deciduous woodlands demonstrating an extensive knowledge base perhaps reflecting the socio-cultural context of relative isolation and long-term settlement of the Vhavenda in the region. The dominance of indigenous plant species represented in ethnobotanical accounts suggests that plant knowledge assimilation may begin with common and readily available plants; however, the recent popularity of exotic tree species also demonstrates a form of adaptation to new introduced species that have become incorporated into the Vhavenda repository of useful plant species. Local practices and institutions are also embedded in cultural contexts and encoded in cosmologies and belief systems that have ensured the sustainable use of plant resources. Plant management strategies aim to sustain reliable and continued supply of plant resources for food, medicine and other uses through selective practices such as the prohibition of certain plants from being used, the promotion of sustainable harvesting practices and the propagation of plant species.

The research highlights the continued importance of indigenous and local knowledge for natural resource management and overcomes assumptions that local knowledge is merely anecdotal or strategic but is dynamic. The Vhavenda hold an extensive array of plant knowledge and intact belief system that is largely determined in the context of social-cultural change by how epistemological and intergenerational frictions are negotiated by individuals and communities and how different knowledge forms are largely accepted, integrated and adapted. The encouragement of hybrid knowledge co-production through the development of collaborations between state-sponsored management, conservation experts, researchers and indigenous and local knowledge holders can lessen the dominance of science and positivism as the primary decision-making frameworks for natural resource management on communal lands. The formation of new partnerships and forums for knowledge exchange between different stakeholders can open the door for more inclusive processes to explore how science and other knowledge systems can align with conservation efforts to enhance the productivity and biodiversity of communal land in South Africa.

## Abbreviations

CBD: Convention of Biological Diversity
ILK: Indigenous and Local Knowledge
IPBES: Intergovernmental Platform on Biodiversity and Ecosystem Services
RFI: Relative Frequency Index
SAPIA: Southern African Plant Invaders Atlas
UVH: University of Venda Herbarium
UVI: Use Value Index
